# Supplementary material for: Tracking the hologenome dynamics in aquatic invertebrates by the holo-2bRAD approach
Source: Commun Biol. 2024 Jul 7;7:827. doi: 10.1038/s42003-024-06509-7 (PMC11228047; doi:10.1038/s42003-024-06509-7)
Supplement: Supplementary file 2 — Supplemental Materials [file 42003_2024_6509_MOESM2_ESM.pdf]

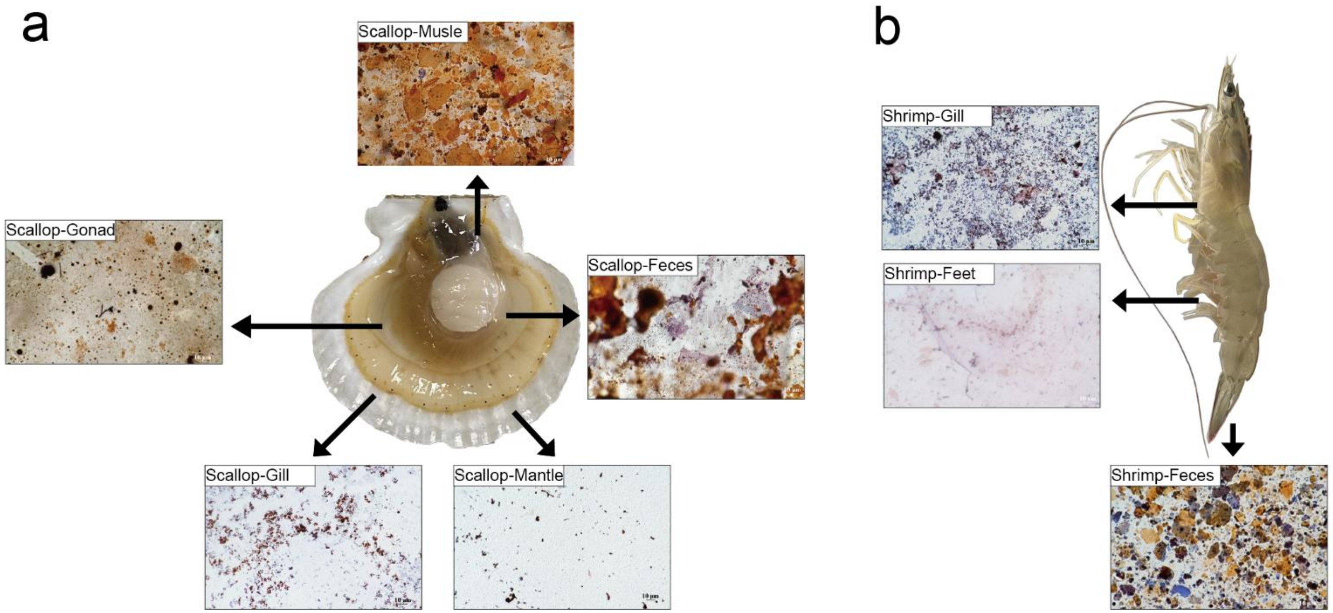

Supplementary Figure 1. Gram staining of different tissues. (a) scallop; (b) shrimp. *The photos of scallop and shrimp were taken by the author Cen Ma.

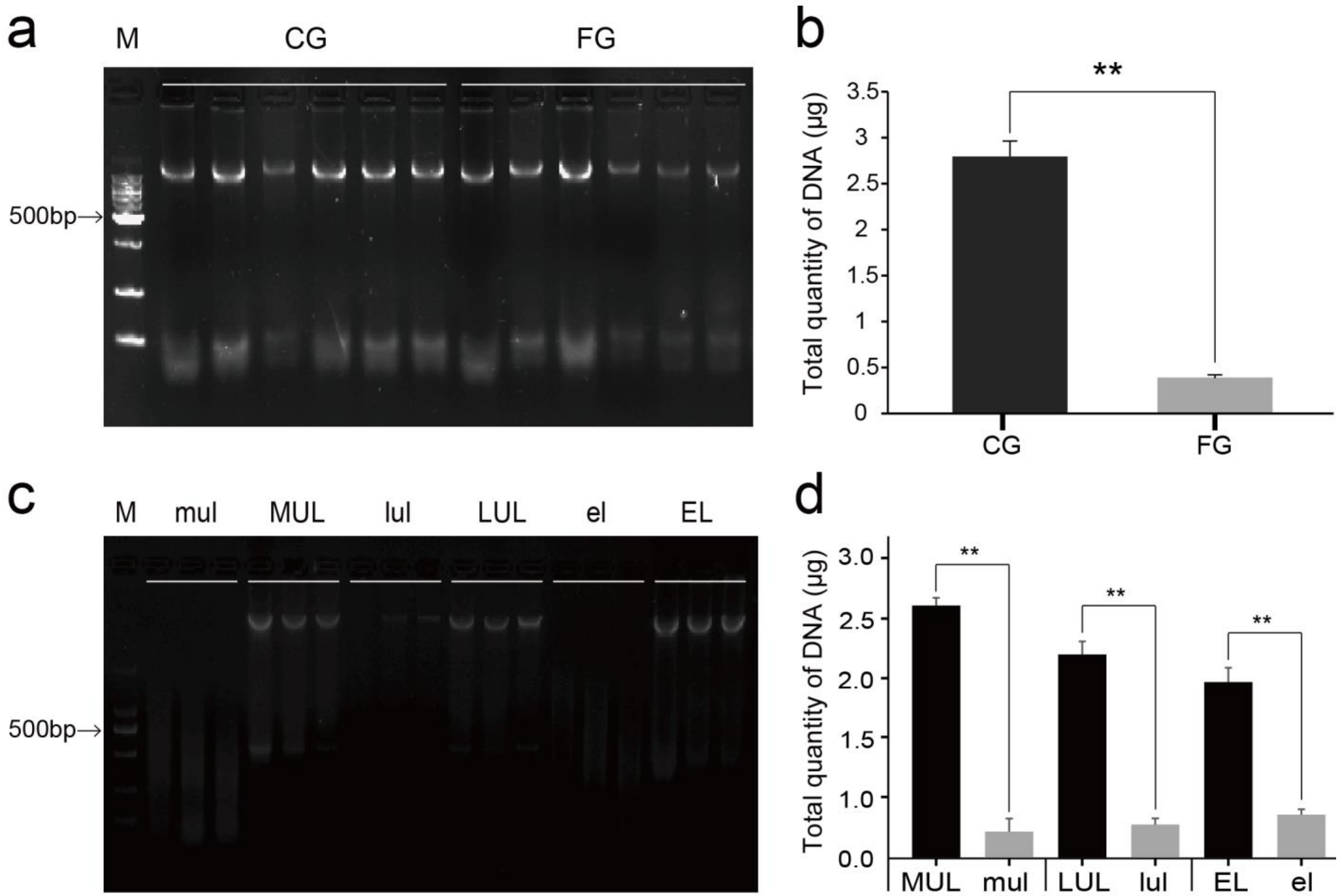

Supplementary Figure 2. Agarose gel electrophoresis analysis of DNA samples. The agarose gel electrophoresis analysis of DNA samples prepared by gill-swabs wiping by cotton swabs (CG), filter paper (FG) (a) and larval samples (c), and DNA yield quantified by spectrometer analysis (after purification) (CG/FG: (b); larval samples: (d)). MUL/mul, middle umbo larvae; LUL/lul, late umbo larvae, EL/el, eyespot larvae (uppercases represent larvae living in the upper layer, lowercases represent larvae living in the bottom layer).

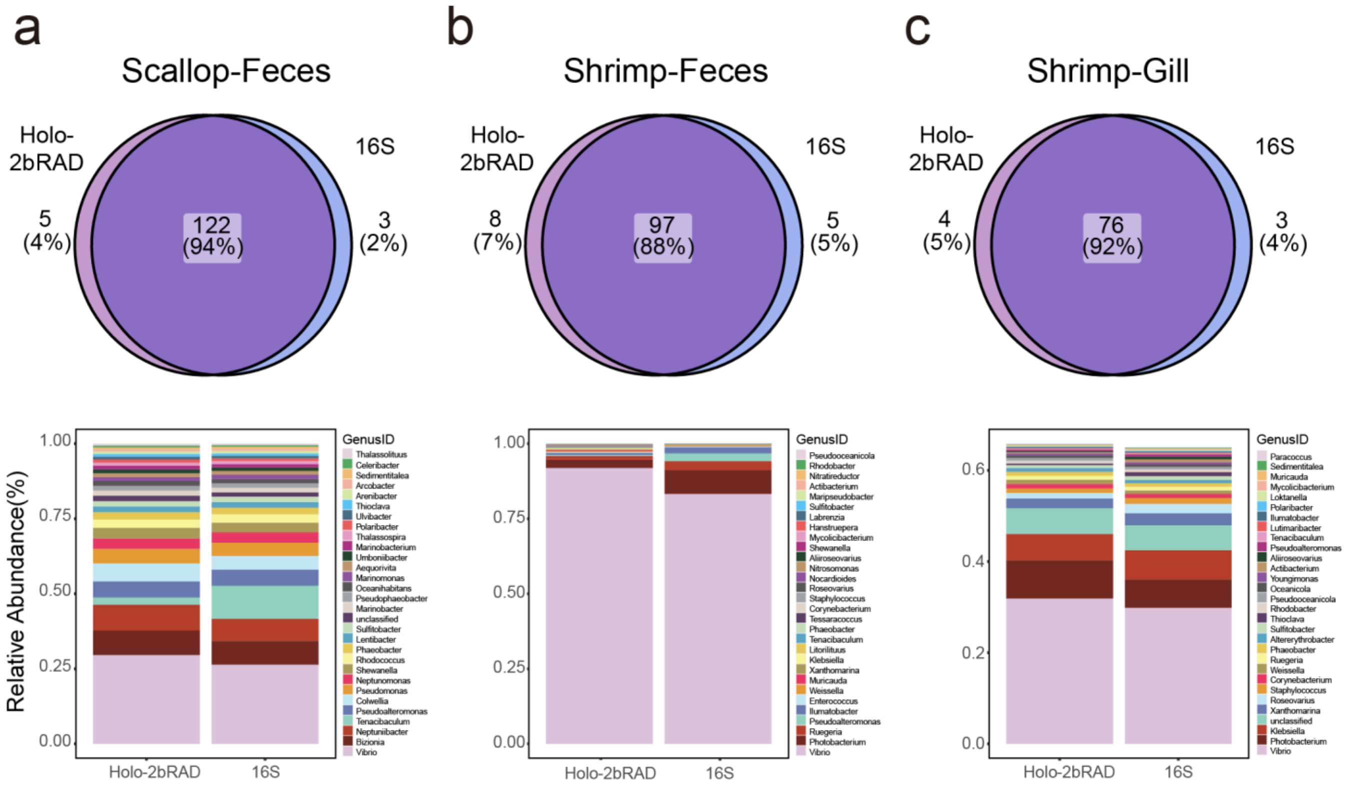

Supplementary Figure 3. The Venn diagram of genus detected and stack column of relative abundance between holo-2bRAD and 16S amplicon sequencing. (a) scallop-feces; (b) shrimp-feces; (c) shrimp-gill.

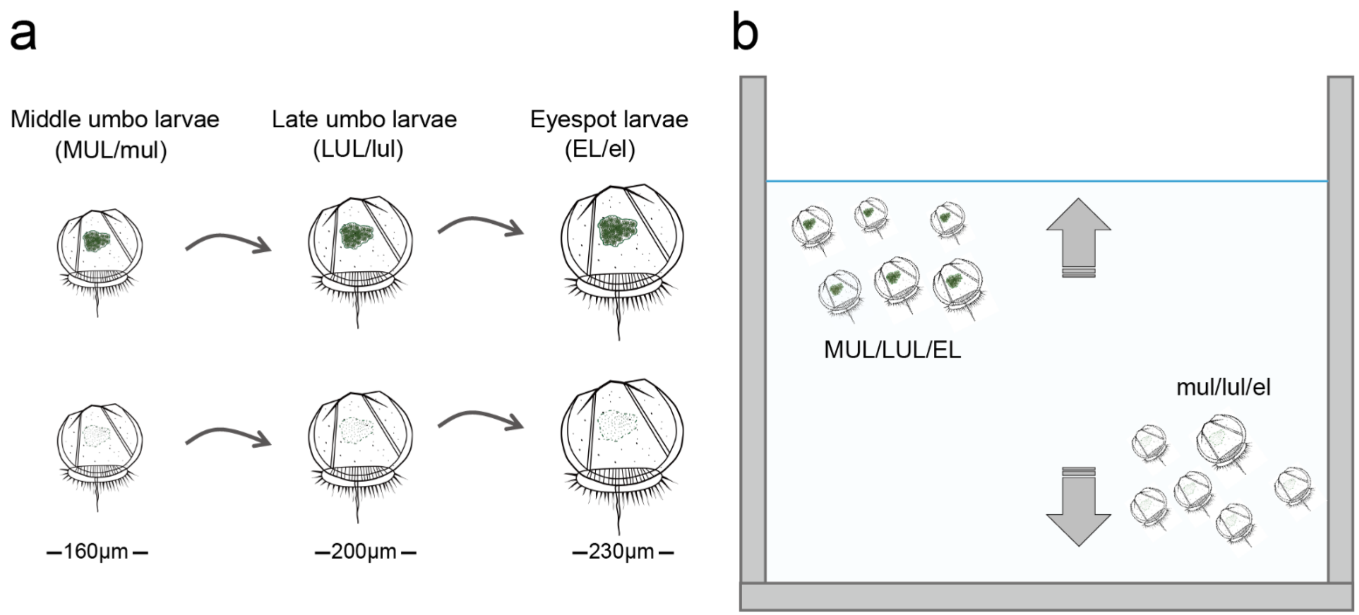

Supplementary Figure 4. Developmental processes and different viability in the larvae of the scallop. (a) developmental processes of larvae; (b) different viability of larvae. MUL/mul, middle umbo larvae; LUL/lul, late umbo larvae, EL/el, eyespot larvae (uppercases represent larvae living in the upper layer with good activity, lowercases represent larvae living in the bottom layer with poor activity).

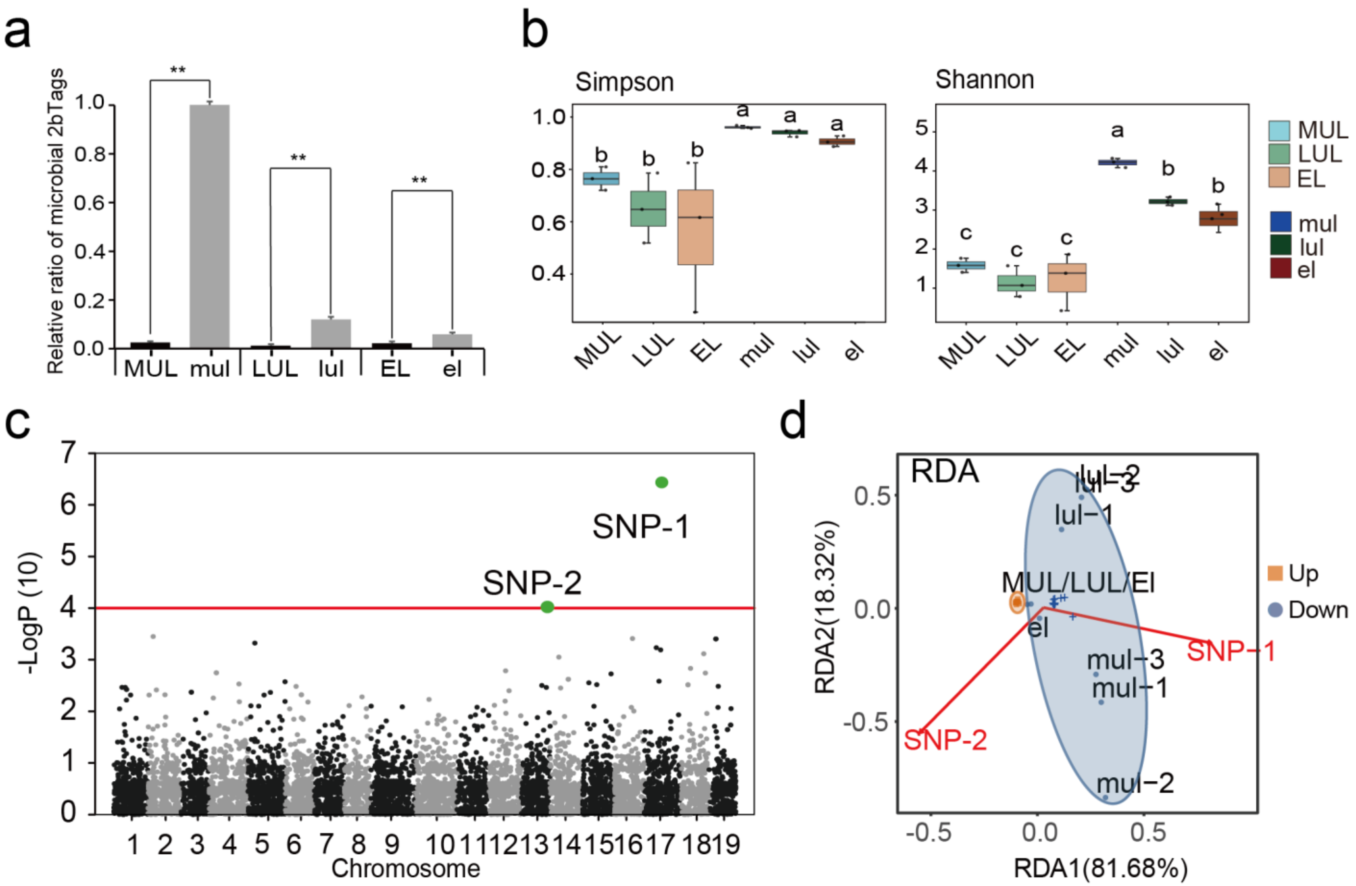

Supplementary Figure 5. Dynamic changes of hologenome in scallop and potential SNPs associated with larval viability. Proportion of DNA reads of scallops and microorganisms (homogenization was performed using the microbial reads detected in mul-1) in MM(a), α- diversity between different groups (b) in MM, Manhattan plots of SNPs detected in a total of 18 samples in MM (c), and RDA analysis of the correlation between two potential SNPs and microbial composition (d). MUL/mul, middle umbo larvae; LUL/lul, late umbo larvae, EL/el, eyespot larvae (uppercases represent larvae living in the upper layer, lowercases represent larvae living in the bottom layer). MM was the culturing hatchery which experienced mass mortalities of larvae.

Supplementary Table 1. Comparison of sites detection and genotype calling between gill/feces and control sample of scallop.

Group	Common detected	Consistent rate (%)	Common calling	Consistent rate (%)
Gill	127,210	93.64	120,632	92.25
Feces	121,166	90.03	119,606	90.17

Supplementary Table 2. Summary of sites detection, genotype calling between replicate libraries of DL/gill/feces of scallop.

		Sites detected	Common detected	Genotype calling	Calling rate (%)	Common Calling
Gill	Rep1	135,932	130,307	123,871	91.13%	120,632
	Rep2	138,893		125,931	90.67%	
	Control	140,848	/	133,468	94.76%	/
Feces	Rep1	132,320	125,870	120,623	91.16%	116,397
	Rep2	132,424		120,467	90.97%	
	Control	139,876	/	132,701	94.87%	/

Supplementary Table 3. Genotyping agreement between replicate libraries of gill/feces of scallop.

		Homozygote	Heterozygote	All
Gill	Genotyped	2,388,753	14,000	2,402,753
	Same genotype	2,386,941	13,453	2,400,394
	Different, genotype	1,812	547	2,359
	Agreement (%)	99.92%	96.09%	99.86%
Feces	Genotyped	2,321,701	14,510	2,346,211
	Same genotype	2,320,019	13,961	2,333,980
	Different, genotype	1,682	749	2,431
	Agreement (%)	99.93%	96.22%	99.90%
